# Immobilizing Laccase on Different Species Wood Biochar to Remove the Chlorinated Biphenyl in Wastewater

**DOI:** 10.1038/s41598-018-32013-0

**Published:** 2018-09-17

**Authors:** Na Li, Qiuyang Xia, Meihong Niu, Qingwei Ping, Huining Xiao

**Affiliations:** 1 Liaoning Province Key Laboratory of Plup and Papermaking Engineering, Dalian Polytech- nic University, Dalian, l16034 China; 2 Key Laboratory of Industrial Ecology and Environmental Engineering, China Ministry of Education, Dalian, 116024 China; 3 Department of Chemical Engineering, University of New Brunswick, Fredericton, NB E3B 5A3 Canada

## Abstract

Biochars produced from two different wood species over a microwave assisted pyrolysis process were used as novel and green-based supports for immobilizing enzyme, laccase in particular. The results obtained from FT-IR, SEM and BET measurements indicated that Maple biochar with honeycomb structure has higher surface area and pore volume than Spruce biochar; and there exist O-H, C-H, C=O and C=C groups in biochars for potential chemical modification. The best laccase immobilization conditions identified from an orthogonal experiment were pH = 3, laccase concentration 16 g/L and contact time 8 h. Under such conditions, the high immobilization yield (64.2%) and amount (11.14 mg/g) of laccase on Maple biochar were achieved, leading to the significantly improved thermal stability of laccase. Moreover, the immobilized laccase is reusable and enhanced the enzymatic degradation of 4-hydroxy-3,5-dichlorobiphenyl (71.4% yield), thus creating a promising and novel type of adsorbent in the removal of polychlorinated biphenyls from wastewater.

## Introduction

Polychlorinated biphenyls (PCBs) belong to persistent organic pollutants (POP)^1^ for their toxicity and stability against degradation in soil and water^2,3^. In the past decades, various technologies have been developed carried out about degradation of PCBs and its bioaccumulation^4,5^. Within all the methods used to degrade the PCBs, laccase was considered as an efficient route to solve the problem because the phenolic compounds were the major substrates of laccase^6,7^, and amount of results showed that the laccase can efficiently remove the PCBs pollutants from the water^8,9^. However, some shortcomings of free laccase prevent its utilization in wastewater treatment, which include instability of thermal and pH, poor reusability and inactivation^10–12^. Enzyme immobilization technique was developed to overcome these negative characteristics, and for its high-efficiency and low-cost, this technique has been considered as one of the most promising methods^13^.

Supports play a very important role in the enzyme immobilization process. Chemically inert polymers and inorganic materials are usually used as carrier matrices^13^. Because enzyme immobilization is a chemical or physical process, the properties of supports could have strong impact on the immobilization yield, enzyme recovered activity, thermal or operation stability, and so on. Besides the economy, other influencing factors, mechanical strength, reusability, ability to promote enzyme thermal and pH stability and non-pollution, should also be considered when choosing support. Therefore, some inorganic and multi-pore materials, such as celite, activated carbon and charcoal, are widely used as supports for enzyme immobilization.

Biochar is the product of heating biomass in the absence of or with limited air to above 250 °C^14^. Normally, the main elements in biochar are C, P, sometimes include some metals such as Ca, Mg or Si. The biochar is chemically stable and has a highly porous structure^15^. According to previous research, biochar is very suitable as adsorbents in environment management over adsorption processes^16,17^; and has been used for the removal of pollutants from soil or water for decades. However, the studies about immobilizing laccase on biochar have been seldom reported. As a novel support for enzyme immobilization, different biochar may present different chemical or physical properties related to different raw materials or production conditions, and this may influence the utilization of biochar for enzyme immobilization. So, two spices wood biochar, one is produced by raw wood chips and the other is produced by commercial wood pellet, were used as support in this study. The enzyme immobilization ability was compared between the two kinds of biochar and the better one was utilized in further research: (1) the thermal and operation stability of immobilized enzyme were studied, (2) the immobilized enzyme was used to degrade the 4-hydroxy-3,5-dichlorobiphenyl (HO-DiCB) and the degradation ability was investigated.

## Results and Discussion

### FT-IR analysis

**Figure 1:**
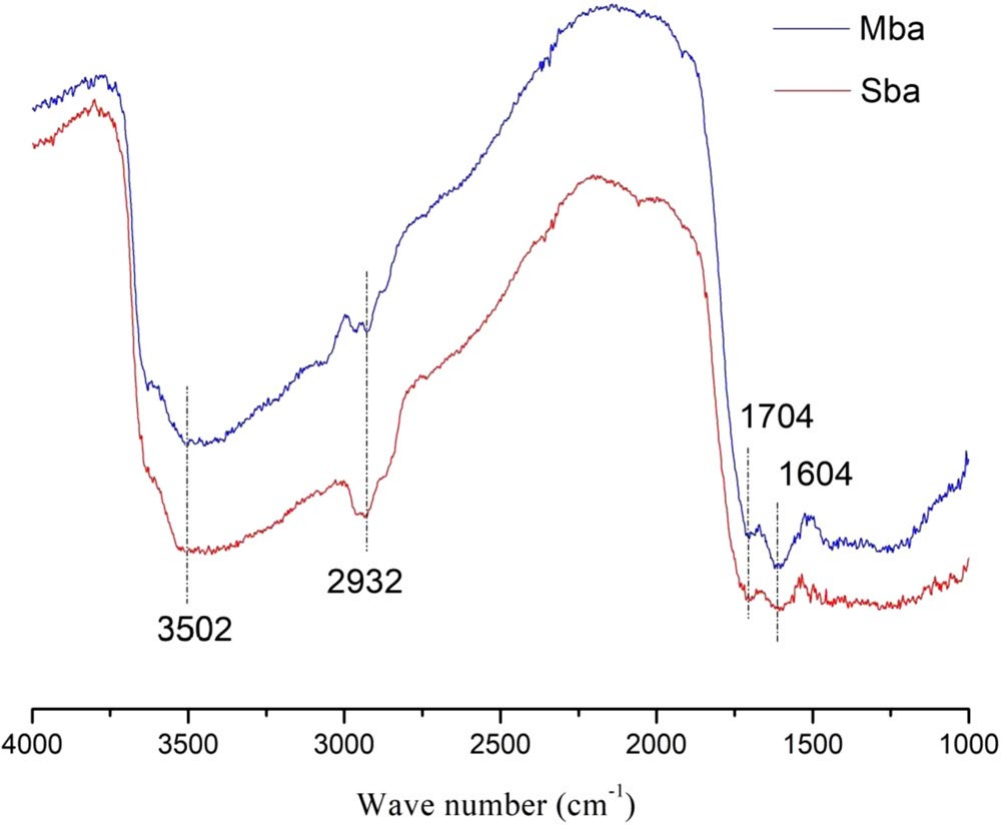
FT-IR of biochar.

Figure 1 shows the FT-IR spectra of biochars. As can be seen from the main peaks of Maple biochar (**Mba**) and Spruce biochar (**Sba**), there are no obvious difference between the two kinds of biochar. The strong band at 3502 cm^−1^ corresponds to O-H and the peak at 2932 cm^−1^ is assigned to C-H group, and the strong absorption at 1704 cm^−1^ and 1604 cm^−1^ are due to C=O and C=C, respectively. The results were consistent with those reported elsewhere using the saligna woodchips as the raw material for biochar^18^. The existence of C-H illustrate that the cellulose was not entirely carbonized during pyrolysis and the C=C bond was assigned to aromatic rings of lignin^18^. The C=O probably derived from ketones, carboxylic acid esters and anhydrides.

### SEM and BET analysis

**Figure 2:**
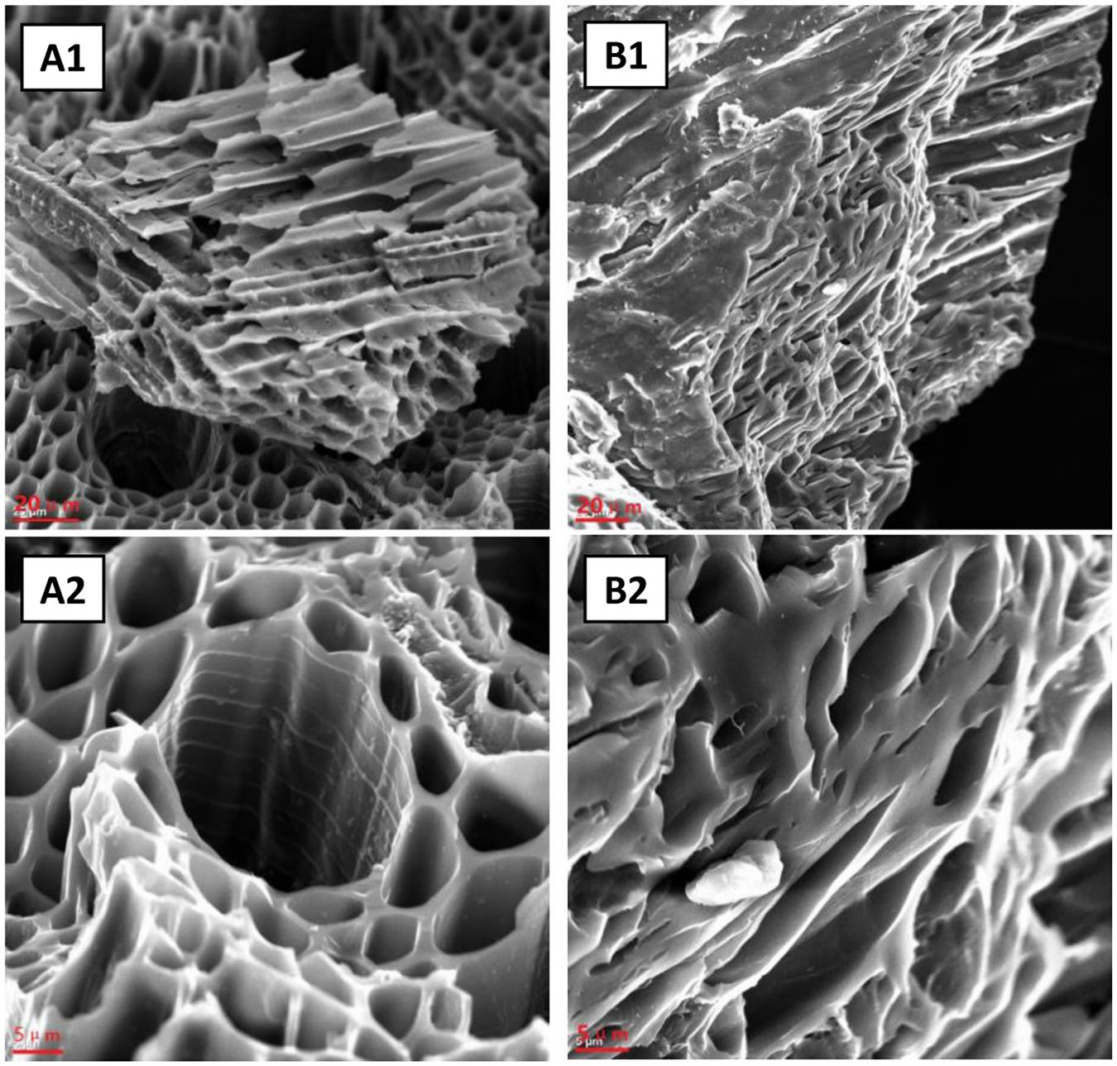
SEM image of biochar, left is Maple sample (A1,A2) and right is Spruce sample (B1,B2).

To observe the microstructure of the two kinds of biochar, SEM images of Maple and Spruce biochars at different magnification were obtained; and the results are shown in Fig. 2. The vessel structure can be seen clearly in both Maple and Spruce biochar images from Fig. 2. Figure 2(A1) shows the vessels of Maple biochar running at different directions in different layers. A magnified image of one striation is shown in Fig. 2(A2), in which a honeycomb structure and regular shape vessels of Maple biochar are visible. Compared to Maple biochar, the running direction of vessels of Spruce biochar appears at single direction, as shown in Fig. 2(B1). Figure 2(B2) shows the shape of vessel is irregular. The difference of the vessel’s arrangement between Maple biochar and Spruce biochar is attributed to different wood species. The shape of the Spruce biochar vessel is more irregular than that of the Maple biochar. In fact, the raw material of Spruce biochar was a kind of compressed particle used for fireplaces; and the compressing process must result in the shape change of the microstructure or collapse of vessels. Contrary to Spruce biochar, the raw materials of Maple biochar are the wood chips from original log; and the original shape of the vessels was well maintained. The SEM results indicated that the microstructures of biochars depended on wood species and post processing on the raw material.

The BET results of Mba and Sba showed (1) the surface areas of Mba and Sba was 613.6 m^2^/g and 86.3 m^2^/g, respectively, (2) the pore volume Mba and Sba was 0.695 cm^3^/g and 0.065 cm^3^/g, respectively. These results demonstrated that the surface area and pore volume of Mba are more superior than those of Sba. Compared to those reported elsewhere, 168 m^2^/g for wood chip biochar produced at 600 °C^19^ and 186 m^2^/g for yellow pine biochar^20^, the surface area of Mba is higher. A larger surface area means more porous structures within biochar^21^. Therefore, the adsorption ability of Mba should be also better than that of Sba.

### Optimum conditions of immobilization

In this study, in order to confirm the best immobilization conditions, an orthogonal experiment was designed. Based on the preliminary study, pH value of the laccase solution, laccase concentration and contact time were chosen as the key variables in the design; and the immobilization yield was selected as the performance target. The parameters used in immobilization and orthogonal design are shown in Table 1 along with the immobilization yields of two kinds of biochar.

**Table 1:** L_16_ (4^3^) orthogonal array design for laccase immobilization on different biochars.

Run	pH	Laccase solution concentration (g/L)	Contact time (h)	Immobilization yield^*^ on Mba (%)	Immobilization yield^*^ on Sba (%)
1	6.00	4.00	4.0	55.41	23.29
2	4.00	8.00	2.0	45.50	28.22
3	5.00	16.00	4.0	38.18	31.10
4	3.00	8.00	4.0	54.84	28.87
5	4.00	16.00	1.0	38.10	30.56
6	6.00	16.00	2.0	24.43	24.06
7	3.00	16.00	8.0	63.71	38.74
8	5.00	32.00	1.0	48.02	28.39
9	5.00	4.00	2.0	36.62	29.57
10	3.00	4.00	1.0	57.48	23.51
11	6.00	8.00	1.0	26.27	29.37
12	4.00	32.00	4.0	30.90	22.40
13	4.00	4.00	8.0	55.85	33.01
14	3.00	32.00	2.0	46.30	27.13
15	5.00	8.00	8.0	42.26	24.86
16	6.00	32.00	8.0	33.86	22.83

^*^Results are mean of ± SD of triplicates.

### Immobilization performance

**Figure 3:**
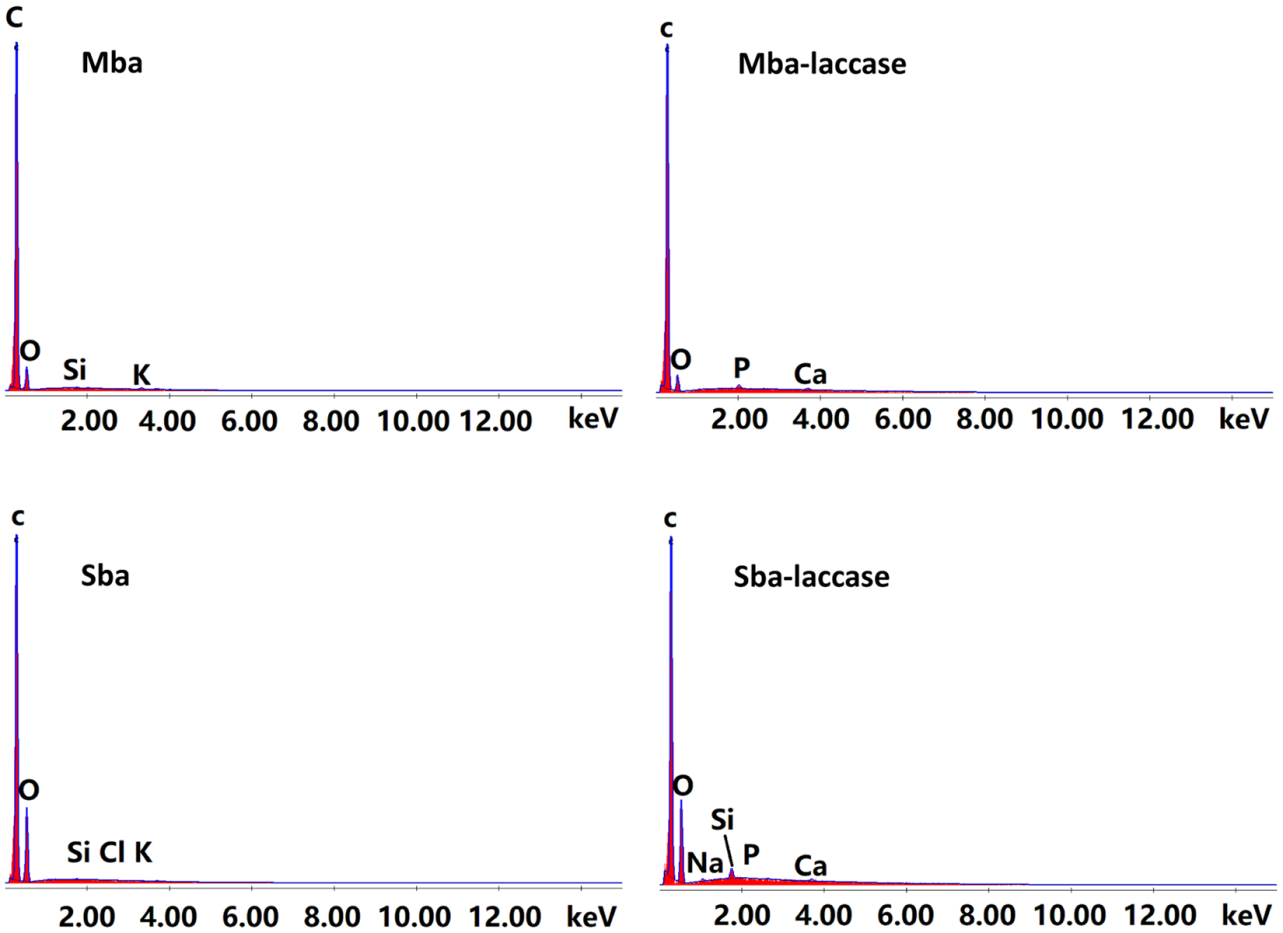
EDS analysis of Mba, Mba-laccase, Sba and Sba-laccase.

According to the orthogonal experiment, pH = 3, laccase concentration 16 g/L and contact time 8 h were chosen to continue the studies. Under these conditions, Mba and Sba were used to immobilize laccase. EDS spectra, shown in Fig. 3, confirmed the existence of laccase on biochar. Compared to Mba and Sba, Mba-laccase and Sba-laccase showed P signal, indicating the presence of the laccase on the Mba and Sba.

The immobilization performances of biochar are presented in Table 2. The immobilization yield and amount of laccase on Mba were obviously higher than those on Sba, suggesting that the adsorption ability of Mba is better than that of Sba. The maximum immobilization yield reached 64.23%; the maximum immobilization amount was 11.14 mg/g Mba. When the activated carbon was used as the support to immobilize laccase, the immobilization yield was 61.43% as reported elsewhere^22^. This result indicated that the performance of the laccase immobilized on biochar was even slightly better than that of conventional activated carbon as support. Laccase immobilized on biochar is a physical process, which is mainly affected by surface area and pore volume. According to BET results, that the surface area and pore volume of Mba are much higher than those of Sba, which allowed the more laccase immobilized on Mba. On the other hand, there are no significant difference between recovered activity of Mba-laccase and Sba-laccase, which illustrated that the recovered activity of immobilized laccase was barely influenced by wood spices of biochar.

**Table 2:** Immobilization performances of Mba and Sba under the same experiment conditions.

Support	Immobilization yield (%)	Immobilization amount (mg/g)	Recovered activity (%)
Mba	64.23	11.14	66.50
Sba	37.62	7.58	63.06

In addition to the surface area and pore volume, the adsorption capacity of biochar is also influenced by biochemical characteristics of different plant biomass, which may yield different physical and chemical properties^23^. The adsorption capacity of grass and straw biochar is much higher than that of hardwood biochar^24^, the copper adsorption capacity of biochar from macro-algae^25^ is ten times higher than that of pine biochar^23,24^^.^ Moreover, the higher adsorption capacity of hardwood compared to softwood was also found in literatures^23^. Overall, due to its superior immobilization performance, Mba was used for laccase immobilization in the rest of the study.

### Stability of immobilized laccase

**Figure 4:**
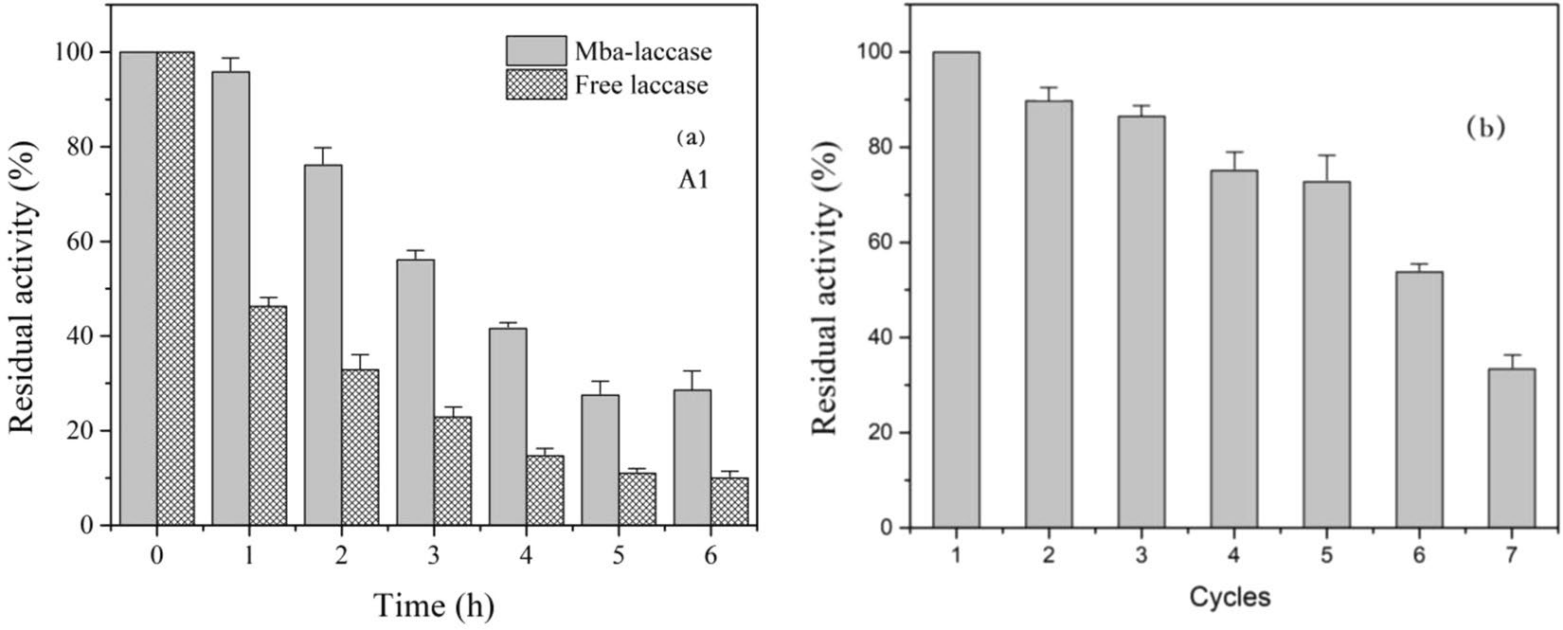
Thermal and operational stability of laccase immobilized on MCCBs by absorption at pH 4.0, 16 g/L initial lacasse concentration and 3 hours contact time.

Two most important parameters related to the application of the immobilized laccase, thermal and operational stabilities were studied. The results are presented in Fig. 4. To evaluate the thermal stability, the residual activities of free and immobilized laccase were measured at 60 °C up to 6 hours. The immobilized laccase was obtained at the optimum experiment conditions: enzyme solution concentration 16 g/L, pH 3.0 and contact time 8 hours. As can be seen from Fig. 4(a), the thermal stability of both the immobilized laccase and free laccase showed a decreased trend, but the thermal stability of immobilized laccase is significantly higher than that of free laccase at 60 °C. At the end of the experiment, 30.3% of the initial activity was maintained for the immobilized laccase; whereas only 10.6% for the free laccase. This result indicate that thermal stability can be improved by immobilization; and the similar results were also reported by others^26,27^. The limited freedom of the immobilized laccase, resulted by immobilization, could decrease the chance of drastic conformational changes and increase the stability of the laccase^28^. Otherwise, immobilization could expose the hydrophobic core of the heated laccase, which makes laccase unlikely to form intermolecular aggregates^29^.

One of the advantages of immobilization laccase is the reusability, which can also affect the cost saving and allow the laccase to be employed in continuous bioreactor operations^30,31^. To identify the reusability, the operational stability was evaluated by 7 cycles’ reaction between immobilized laccase and ABTS. The results are shown in Fig. 4(b). After 7 cycles, the immobilized laccase lost about 66.2% of initial activity. The loss of the activity was probably attributed to: (1) part of immobilized laccase with weak binding desorbed from the Mba support during the washing process for the instability of physical adsorption, (2) the enzyme activity may lost during storage^32^. In this study it took five days to finish the operational stability experiment since the reaction between immobilized laccase and ABTS was slow and continuously slowed down with prolonged the measurements.

### Kinetic parameters of the free and immobilized laccase

**Figure 5:**
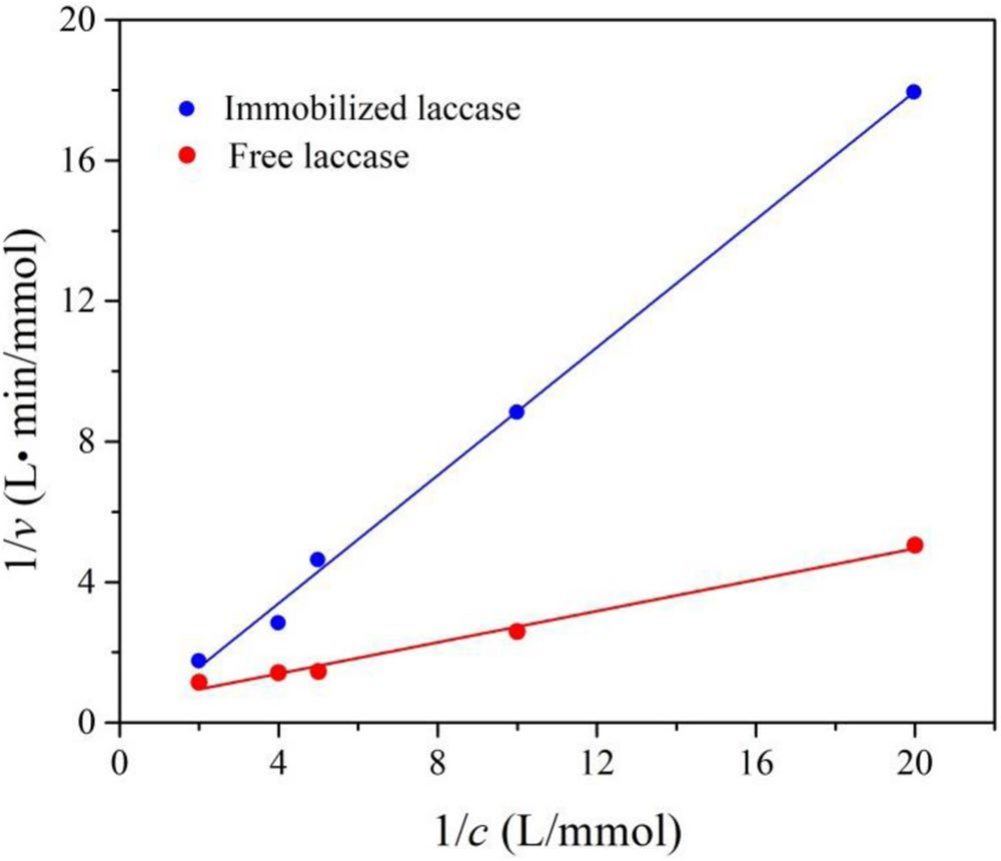
Lineweaver-Burk plots of free and immobilized laccase.

The lineweaver-Bruk plots of free and immobilized laccase are shown in Fig. 5. The immobilized laccase exhibited a higher *K*_m_ than the free one, which are 2.68 and 0.223, respectively. The higher *K*_m_ value, the lower the affinity of the laccase. The lowering of affinity after immobilization is mainly due to the fact that the immobilized laccase is not as free as the free laccase which can fully contact with the substrate. This result is also coincide with the conclusion reported by others, i.e., the immobilization could result in a loss of enzyme activity^33^.

### Degradation of HO-DiCB

**Figure 6:**
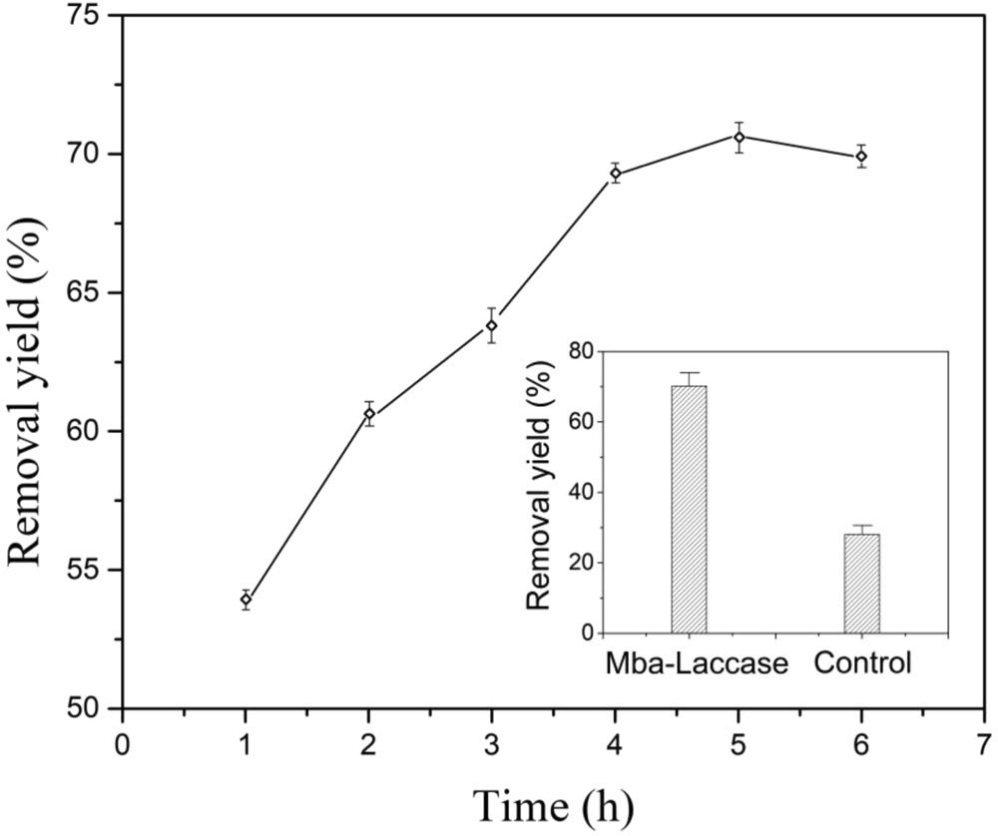
Removal yield of HO-DiCB by Mba-laccase and Control at pH 3.0 room.

The removal yield of HO-DiCB by Mba-laccase is shown in Fig. 6. After 5 h, the removal yield of HO-DiCB by Mba-laccase reached as high as 71.4%. However, the result obtained in the control experiment under the same conditions was only 28.3%.

According to previous studies that the biochar can be used as adsorbent, so the degradation in the control experiment was due to the adsorption of HO-DiCB on Mba instead of biodegradation. The concentration of the HO-DiCB decreased because of the migration of the HO-DiCB from the solution to the surface of the control sample^34,35^. The higher removal yield obtained by Mba, compared to control, indicated that the immobilized laccase can degrade the HO-DiCB effectively. Moreover, considering the removal ability of the control Mba, the high removal rate of the immobilized laccase is attributed to both enzymatic degradation and adsorption.

## Conclusions

Two kinds of wood biochar were successfully used as a novel type of support for immobilizing laccase to promote the enzymatic degradation of HO-DiCB. The biochars prepared via a microwave assisted pyrolysis process possess proper porous structures. Maple-based one in particular, which showed much better performance than Spruce biochar in terms of the immobilization yield and the recovered activity of laccase. The immobilization improved the thermal stability of laccase and induced the enzymatic degradation of HO-DiCB effectively, thus leading to a reusable, cost-effective and green-based adsorbent which is highly promising in the removal PCBs from wastewater.

## Materials and Methods

### Materials

The following reagents were all purchased from Sigma chemical (Canada): anhydrous citric acid, Na_2_HPO_4_·12H_2_O, coomassie brilliant blue (CBB), 95% ethyl alcohol, 85% H_3_PO_4_, NaCl, bovine serum albumin (BSA), sodium hydroxide and 2,2′-azino-bis (3-ethylbenzothiazoline-6-sulfonic acid) (ABTS). HO-DiCB was purchased from AccuStandard, USA. Biochars were kindly provide by Prof. Afzal’s group at the University of New Brunswick.

Laccase (800 U/g) was kindly donated by Chongqing Saipunasi Science and Technology Ltd. The laccase was produced from coprinus comatus (mushroom) and the culture conditions were: pH = 4, 25 °C, 320 rpm, original inoculum concentration (OD600) 30, 400 uM Cu^2+^, 0.5% casamino acid and 1.5% methyl alcohol. The purification of the enzyme was performed using DEAE-sepharose CL-6B ion exchange column, followed by Sephadex G-75 molecular sieve chromatography. The storage and delivery temperature was −20 °C.

### Preparation of biochar

Two kinds of raw materials were used to produce biochars in this study, which were Maple and Spruce wood pellet, belonging to hard and soft wood, respectively. The biochars were prepared over a microwave assisted pyrolysis process^36^. The resulting biochars were mechanically ground into fine particles; and those with size between 20–45 mesh were collected and used as the supports and adsorbents, then the two kinds of biochar adsorbents, Maple biochar adsorbent (Mba) and Spruce biochar adsorbent (Sba) were obtained.

### Characteristics of biochar

The chemical groups of Biochar were analyzed by FTIR. A scanning electron microscopy (SEM) (JEOL 6400, JEOL, Japan) was used to observe the surface morphology of the Mba and Sba. The specific surface area, average pore size and volume of biochar fine particles were determined with BET measurements using an autosorb instrument (Belsorp-Max BEL Inc, Osaka, Japan).

### Determination of laccase activity

The enzyme activity of free and immobilized laccase were measured by reacting with 0.4 mM ABTS solution at 25 ± 1 °C^37^, and a UV-Vis spectrophotometer (Genesys 10-S, Thermo Electron Corporation, USA) was used in this study. The measurement of free enzyme activity is detailed as follows: (1) 0.1 mL of free laccase solution was added into 1.9 mL of ABTS solution, (2) recorded the UV absorbance of the mixture solution at 420 nm (ε420 = 36000 M^−1^ cm^−1^) per 30 seconds and persisted 5 minutes, (3) form a kinetic curve based on the recorded data and measure the slope of initial linear portion of the curve. Unit of free enzyme activity was expressed in U/L and one U was defined as the amount of free enzyme required to catalyze 1 μmol of substrate per minute.

The detailed measuring operation of immobilized enzyme activity was: (1) 0.1 g support-laccase was put in 7.0 mL of citrate-phosphate buffer and 2 mL of ABTS mixture solution, (2) in order to record the UV absorbance of the mixture solution, 2 mL of mixture solution was withdrawn as a simple to analysis absorbance at 420 nm per 2 minutes, and the sample was put back to reactor rapidly after analysis, (3) form a kinetic curve based on the recorded data and measure the slope of initial linear portion of the curve. Unit of immobilized enzyme activity was expressed in U/g.

### Immobilization of laccase

To study the main effect factors on immobilizing laccase on biochar, a L_16_ (4^3^) orthogonal experiment was designed and analyzed by SPSS software. Based on the orthogonal experiment results, a set of conditions, including pH value, contact time and laccase solution concentration, were chosen over the immobilization experiments.

An electron dispersive spectroscopy (EDS) was used to perform an elemental analysis of the Mba-Laccase and Sba-Laccase with 15 kV (JEOL 2011, JEOL, Japan).

The immobilization yield was calculated as follows:

1 $$\begin{array}{c} \% \,{\rm{immobilization}}\\ \,=\,\frac{Original\,enzyme\,concentration-Enzyme\,concentration\,after\,immobilization}{Original\,enzyme\,concentration}\times 100 \% \end{array}$$

Measurement of enzyme concentration referred to Bradford method^38^.

The recovered activity was calculated using the following equation:

2 $$ \% \,{\rm{recovered}}\,{\rm{activity}}=\frac{immobilized\,enzyme\,activity\,}{activity\,of\,same\,amount\,of\,free\,enzyme}\times 100 \% $$

### Thermal and operational stability of immobilized laccase

To evaluate the thermal stability, the enzyme activities of the free and immobilized laccase were measured in pH 4.0 butter at 60 °C every 1 hour and the experiment lasted 6 hours. A water bath with temperature controller was used in this experiment. The initial activity was set as 100%.

0.1 g laccase-support was used to measure the operational stability in 2 mL of ABTS and 7 mL of citrate-phosphate buffer at pH 4.0 and 25 ± 1 °C conditions. The stability experiments were operated for 7 cycles. The immobilized laccase enzyme activity was measured at each cycle and the laccase-support was separated by filtration and washed twice with 30 mL of buffer solution before next cycle.

### Determination of kinetic parameters

To determine the kinetic parameters *K*_*M*_ of free and immobilized laccase, the enzyme activities were measured by using ABTS as substrate which initial concentrations were arranged from 0.05 mM to 1.0 mM. The kinetic parameters were obtained by non-linear curve fitting of the plot of reaction rate versus substrate concentration, in which the 1/*c* (*c*, concentration of the substrate) and the 1/*v* (*v*, enzyme initial reaction rate) were set as X-axis and Y-axis, respectively.

### Degradation of HO-DiCB by immobilized laccase

A HO-DiCB aqueous solution was prepared by introducing 10 mg HO-DiCB reacted with 1.7 mg NaOH in 50 mL of deionized water and adjusting the final volume to 250 mL in volumetric flask and the finale concentration of HO-DiCB was 0.04 g/L. To evaluate the degradation yield of the HO-DiCB, 50 mg Mba-laccase was added into 10 mL of HO-DiCB aqueous solution and 10 mL of pH 3.0 phosphate buffer. The concentration of HO-DiCB was measured each hour in order to calculate the degradation yield, and the whole experiment was finished at the sixth hour for the slight change of HO-DiCB concentration. The concentration of HO-DiCB aqueous solution was determined by an ACQUITY UPLC H-Class PLUS system (Waters) equipped with a C18 Colume (100-2.1 mm; Waters). Mobile phase was 50% (vol) acetonitrile in aqueous solution at a flow rate of 0.5 ml/min. The detection wavelength was 260 nm. Degradation yield (%) was defined as follows:

3 $$ \% \,{\rm{degradation}}=\frac{original\,HO-DiCB\,amount-final\,HO-DiCB\,amount}{original\,HO-DiCB\,amount}\times 100 \% $$

At the same time, 50 mg Mba-laccase adsorbed with inactive laccase was added into 10 mL of HO-DiCB aqueous mentioned above to conduct the control experiment under the same conditions.
